# Segmentation of Hyperacute Cerebral Infarcts Based on Sparse Representation of Diffusion Weighted Imaging

**DOI:** 10.1155/2016/2581676

**Published:** 2016-09-22

**Authors:** Xiaodong Zhang, Shasha Jing, Peiyi Gao, Jing Xue, Lu Su, Weiping Li, Lijie Ren, Qingmao Hu

**Affiliations:** ^1^Shenzhen Institutes of Advanced Technology, Chinese Academy of Sciences, 1068 Xueyuan Boulevard, Shenzhen 518055, China; ^2^Beijing Tiantan Hospital, Capital Medical University, 6 Tiantan Xili, Beijing 100050, China; ^3^Shenzhen Second People's Hospital, 3002 West Sungang Road, Shenzhen 518035, China

## Abstract

Segmentation of infarcts at hyperacute stage is challenging as they exhibit substantial variability which may even be hard for experts to delineate manually. In this paper, a sparse representation based classification method is explored. For each patient, four volumetric data items including three volumes of diffusion weighted imaging and a computed asymmetry map are employed to extract patch features which are then fed to dictionary learning and classification based on sparse representation. Elastic net is adopted to replace the traditional *L*_0_-norm/*L*_1_-norm constraints on sparse representation to stabilize sparse code. To decrease computation cost and to reduce false positives, regions-of-interest are determined to confine candidate infarct voxels. The proposed method has been validated on 98 consecutive patients recruited within 6 hours from onset. It is shown that the proposed method could handle well infarcts with intensity variability and ill-defined edges to yield significantly higher Dice coefficient (0.755 ± 0.118) than the other two methods and their enhanced versions by confining their segmentations within the regions-of-interest (average Dice coefficient less than 0.610). The proposed method could provide a potential tool to quantify infarcts from diffusion weighted imaging at hyperacute stage with accuracy and speed to assist the decision making especially for thrombolytic therapy.

## 1. Introduction

Irreversible infarcts are critical for the assessment of potential risk and benefit pertaining to thrombolysis in hyperacute ischemic stroke [1]. Due to the high sensitivity and specificity of diffusion weighted (DW) imaging (which consists of T2-weighted image (*b* = 0) to be denoted as B0, a diffusion weighted image (DWI) with the *b* value being 1000–1500 s/mm^2^, and the calculated apparent diffusion coefficient (ADC) map), it is considered the optimum clinical imaging modality for hyperacute ischemic stroke [2]. Previously, it was reported that DW imaging reversal was not rare [3], putting a question mark on determination of infarcts from DW imaging. Recently, it has been found that the DW imaging reversal is rare [1] and does not translate to permanent tissue salvage [4]. This new finding justifies the urgent need for accurate determination of infarcts from DW imaging.

**Figure 1:**
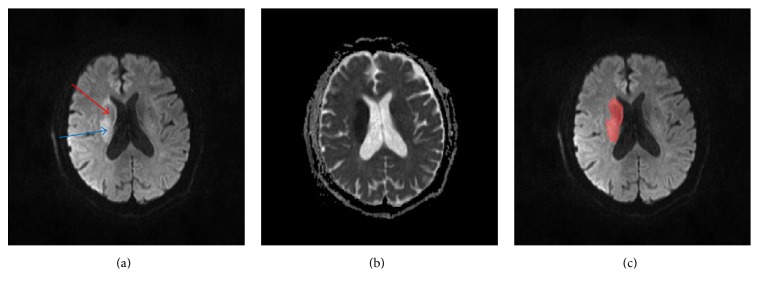
Illustration of inhomogeneous ischemic lesions. From (a) to (c): an axial slice of DWI with inhomogeneous infarcts (red and blue arrows, resp., pointing to hyperintense and isointense DWI regions of the lesion); the corresponding ADC and the ground truth lesion (in red) overlapped on the DWI slice.

Ischemic lesions are inhomogeneous in terms of ischemic injury and recovery potential [5] and could be classified into 4 categories on DWIs [6]: single lesion with well-defined edges, single lesion with ill-defined edges, multiple lesions with well-defined edges, and multiple lesions with ill-defined edges. It has been shown that lesions at hyperacute stage (i.e., within 6 hours from ictus) exhibit greatest variability in appearance (Figure 1) which makes expert delineation difficult and inconsistent [6]. The difficulties in identifying infarcts at hyperacute stage were also reflected in an automatic method to segment infarcts from DWIs, where the segmentation was substantially less accurate for patients imaged at the time of admission than for those imaged at 72 hours (mean Dice coefficient (DC) of 0.63 versus 0.81) [7].

There have been efforts on automatic segmentation of infarcts from DW imaging. Tsai and coworkers segmented infarcts from DWIs and ADC maps based on fuzzy C-means (FCM) clustering [8]: it used the most frequent normalized DWI intensity *I*_peak_ within the brain to remove nonrelevant voxels, classified the remaining voxels into 50 clusters, removed clusters and connected components whose average DWI intensity was not greater than *I*_peak_ + 0.2, eliminated false positive regions without apparent edges, and got rid of false positive regions due to magnetic inhomogeneity by imposing ADC constraints. It reported an average DC of 0.899 ± 0.065 for 22 ischemic patients with stroke onset within 10 days. Mujumdar et al. [9] segmented infarcts from DWIs through 3 *b* values (*b* = 0, 1000, and 2000 s/mm^2^): multiple *b* values were employed to impose local contrast constraints; the left candidates were passed to an active contour model to refine segmentation. It reported an average DC of 0.810 ± 0.120 for 41 ischemic stroke patients without specifying stroke onset time. Prakash et al. [7] segmented infarcts from DWIs by first identifying axial slices with ischemic lesions followed by binarization with a global DWI intensity threshold derived from histogram divergence: it was basically a global thresholding method based on the assumption of asymmetry induced by the ischemic lesion; it was tested on 57 datasets with 46 scanned at the time of admission without knowing the exact imaging time and 11 scanned at 72 hours from admission, to yield an average DC of 0.670 ± 0.220.

As images are naturally sparse and have redundant information, sparse representation has been widely used in image processing [10]. The first successful application of sparse representation to computer aided diagnosis was by Liu and coworkers [11]. Their method was extensively validated for both colorectal polyp and lung nodule detection and could achieve superior classification/segmentation performance to existing methods using support vector machine and its variants, boosting, logistic regression, relevance vector machine, or *k*-nearest neighbors. The success of sparse representation based classification/segmentation owes to the fact that a high-dimensional image can be represented or coded by a few representative samples from the same class in a low-dimensional manifold and the recent progress of *l*_0_-norm and *l*_1_-norm minimization technique [12]. Sparse representation could be used as a classifier for voxel-wise classification. Zhang et al. [13] made use of sparse representation to segment cervigram images. Based on traditional constructive dictionary learning method, they proposed a discriminative dictionary learning method. The learned discriminative dictionaries were more suitable for classification and achieved better performance to segment tissues in optical images of the uterine cervix. A prostate segmentation framework based on sparse representation was proposed by Gao et al. [14]. Different from conventional dictionary learning method, discriminative dictionaries were learned through k-means after feature selection. New samples were classified according to reconstruction error computed with the learned discriminative dictionaries. It performed better to segment prostate in computed tomography (CT) images compared with other state-of-the-art methods.

Sparse representation has also been incorporated into atlas-based methods for image segmentation. Wang et al. [15] used sparse representation to build subject-specific atlas from a series of aligned atlases. The subject-specific atlas built based on the reconstruction coefficient was integrated into a level set framework for further accurate segmentation. Following the same principle, Wang et al. [16] built a patient-specific atlas from the patch-based sparse representations for tissue segmentation of cone-beam CT images. Then the built atlas was integrated into a maximum a posteriori probability-based convex segmentation framework for accurate segmentation.

There are other applications of sparse representation in the field of medical image processing. Fang et al. [17] proposed a sparse perfusion deconvolution method to estimate cerebral blood flow in CT perfusion at low radiation dose. The sparse dictionary was built from high-dose perfusion maps. Then the built dictionary was applied to low-dose data to perform deconvolution-based hemodynamic parameters estimation.

On DWIs, infarcts appear as hyperintense and inhomogeneous in the form of intensity variation, with complex shapes and ambiguous boundaries, which makes manual segmentation difficult, time consuming, and rater dependent. As time is critical for hyperacute ischemic stroke patients, especially for those who are potential candidates to have thrombolytic therapy, it is highly desirable to quantify their infarcts with accuracy and speed. To the best of our knowledge, on the one hand, segmenting infarcts based on sparse representation has not been reported; on the other hand, efforts on segmenting hyperacute infarcts with great variability are scarce. These two issues are to be investigated in this study.

## 2. Sparse Representation Based Classification

### 2.1. Sparse Representation

Sparse representation is a powerful tool for acquiring, representing, and compressing signals. Given a dictionary, it selects only a few elements in the dictionary under certain constraints to reconstruct best the signal through linear combination of the selected elements. Suppose a dictionary *D* ∈ *R* ^*m*×*K*^ with  *K* elements of *m* dimensions; sparse representation of a signal *s* ∈ *R* ^*m*×1^ is formulated as follows:

(1) α^s,D=arg⁡minα∈RK s−Dα22,s.t. α0≤λ,

where *α* ∈ *R* ^*K*^ is the sparse code or sparse vector, ‖·‖_0_ is *L*_0_-norm, and  *λ* is the sparsity which constrains the number of nonzero elements in the sparse vector *α*.  Formula (1) is optimized to find the optimal sparse code that leads to lowest reconstruction error with a fixed sparsity *λ*.

The dictionary *D* ∈ *R* ^*m*×*K*^ is generally attained through a dictionary learning process. Given a set of training signals *s*_*i*_ ∈ *R* ^*m*×1^,  *i* = 1,2 …, *n*, the dictionary *D* is constructed such that the following conditions are met:

(2) minα,D ∑i=1nsi−Dαi22,s.t. α0≤λ.

It aims at finding the optimal dictionary *D* that best reconstructs input training signals under *L*_0_-norm constraint on sparse code *α*_*i*_ ∈ *R* ^*K*×1^ of signal *s*_*i*_. The objective function can be optimized by several algorithms such as K-SVD [18] or MOD [19].

As the objective functions defined in formulae (1) and (2) are nonconvex and nonsmooth, finding the solution is NP-hard. *L*_1_-norm could be used as a convex relaxation to replace *L*_0_-norm:

(3) α^s,D=arg⁡minα∈RK s−Dα22,s.t. α1≤λ

(4) minα,D ∑i=1nsi−Dαi22,s.t. α1≤λ,

where *λ* is a parameter that controls the sparsity of sparse vector *α* and ‖·‖_1_ is *L*_1_-norm. It can be treated as the LASSO problem and solved effectively by LARS [20].

### 2.2. Sparse Representation Based Classification

Sparse representation based classification (SRC) has been employed in the pattern recognition field and achieved state-of-the-art results in areas like human face recognition [21]. SRC consists of two stages. At first, given *n* training samples and *C* labels

(5) $$\begin{array}{c} \left\{\;\left(\;s_{i},l_{i}\;\right)\;|\;s_{i}\in R^{m\times 1},\;\;l_{i}\in \left[\;1,2,\ldots ,C\;\right],\;\;i=1,2,\ldots ,n\;\right\}. \end{array}$$

Subdictionaries {*D*_*i*_ ∈ *R* ^*m*×*K*_*i*_^∣*i* = 1,2,…, *C*} are learned with corresponding samples in every class by formula (2) or (4), with *K*_*i*_ being the size of dictionary *D*_*i*_.

In the second stage, when a new sample *s* ∈ *R* ^*m*×1^ is given to be classified, the global dictionary *D* = [*D*_1_, *D*_2_,…, *D*_*C*_] ∈ *R* ^*m*×∑_*i*=1_^*C*^ *K*_*i*_^ is used to sparsely represent *s* in a competitive manner to select basis elements. The reconstruction error of every class is

(6) $$\begin{array}{c} r_{i}={\left\|\;s-D_{i}\delta_{i}\left(\;\hat{\alpha }\;\right)\;\right\|}_{2}^{2},\;i=1,2,\ldots ,C, \end{array}$$

where *δ*_*i*_(·) is a characteristic function that selects the coefficients associated with the *i*th class (*D*_*i*_). Then the new sample is classified into the class with the lowest reconstruction error.

The SRC framework could be employed for medical image segmentation. However, different from face recognition, medical images are inherently three-dimensional (3D) to be computationally demanding. In addition, there could be strongly correlated samples in the object and background such that the learned subdictionaries could contain strongly correlated basis elements which make the sparse code unstable. We explore the extension of SRC for segmenting cerebral infarcts from DW imaging to be elaborated next: to reduce the computational cost by confining the searching space and to avoid the unstable sparse coding by replacing the *L*_0_-norm/*L*_1_-norm with elastic net.

## 3. Infarct Segmentation Based on Sparse Representation

**Figure 2:**
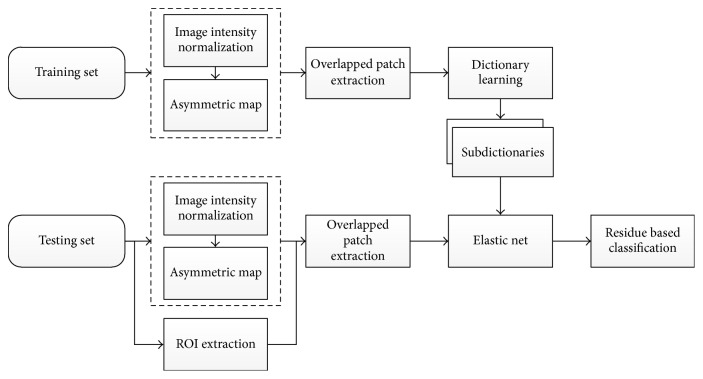
Flow chart of the proposed method, ROI for region-of-interest.

The proposed method extends the SRC framework to segment cerebral infraction (Figure 2). It consists of dictionary learning and voxel-wise classification. For a patient, in addition to the 3 volumes of DW imaging, a new volume is calculated to represent the asymmetry feature due to infarction. Local patches of the 4 volumes are employed to extract patch features. During voxel-wise classification, regions-of-interest (ROIs) are extracted based on expansion of the ischemic regions that have been validated previously [22]. Only voxels within ROIs are considered as candidates for classification based on elastic net.

Denote the baseline B0, DWI, and ADC images as B0(*x*, *y*, *z*), DWI(*x*, *y*, *z*), and ADC(*x*, *y*, *z*), respectively. The algorithm can be decomposed into preprocessing, feature extraction, dictionary learning, derivation of ROIs, and classification.

### 3.1. Preprocessing [23]

The original volumes can have large intensity range which is rescaled to [0,255] to facilitate subsequent processing.

The 3 volumes B0(*x*, *y*, *z*), DWI(*x*, *y*, *z*), and ADC(*x*, *y*, *z*) all play a role in differentiating infarct voxels from noninfarct voxels. Specifically, hyperintense B0(*x*, *y*, *z*) could be used for differentiating fresh from old infarction; an infarct will have hyperintense DWI(*x*, *y*, *z*) and hypointense ADC(*x*, *y*, *z*). A volume DWI_ADC(*x*, *y*, *z*) can be generated to emphasize infarcts.

(7) $$\begin{array}{c} DWI\_ADC\left(\;x,y,z\;\right)=\left\{\begin{array}{cc} DWI\left(x,y,z\right)-ADC\left(x,y,z\right), & \text{if }DWI\left(x,y,z\right)-ADC\left(x,y,z\right)>0\\ 0, & \text{otherwise.} \end{array}\right. \end{array}$$

**Figure 3:**
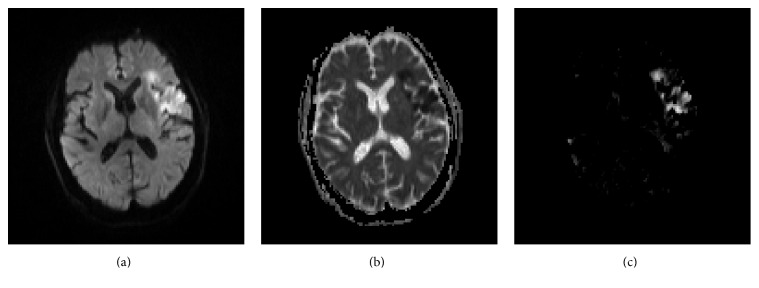
From (a) to (c), an axial slice of DWI and ADC and corresponding asymmetry map ASYM.

As infarction is generally asymmetrical with respect to the midsagittal plane (MSP), a composite volume is formulated and is denoted as ASYM(*x*, *y*, *z*) in the following way:

(8) ASYMx,y,z=difx,y,z,if difx,y,z>00,otherwise,difx,y,z=DWI_ADCx,y,z−maxu,v∈Nsx0,y0⁡DWI_ADCu,v,z,

where (*x*_0_, *y*_0_) and (*x*, *y*) are symmetrical to the midsagittal line (which is the intersection between the MSP and the axial slice *z*) on the axial slice *z* and *N*_*s*_(*x*_0_, *y*_0_) is the neighborhood of (*x*_0_, *y*_0_) and is set as 5*∗*5 neighborhood. MSP is extracted from B0(*x*, *y*, *z*) based on local symmetry and outlier removal [24].  Figure 3 shows an axial slice of DWI, ADC, and ASYM.

### 3.2. Feature Extraction

Based on the fact that image patches could capture more anatomical information than a single voxel, patch-based methods have been recommended for label fusion and segmentation [10]. For every sample voxel, four patches centered at the voxel with size *d* are obtained from volumes B0, DWI, ADC, and ASYM. Then intensity values of each patch are rearranged into a column-vector. These column-vectors are concatenated into the final feature vector.

In this study, both two-dimensional (2D) and 3D patches will be explored. Square patches are obtained from axial slice of four volumes to form a 4*∗d* ^2^ feature vector for 2D patches; cuboid patches are obtained from four volumes and concatenated into a 4*∗d* ^3^ feature vector for 3D patches. Here *d* = (2*∗R*_*p*_ + 1) with *R*_*p*_ = 1,2, 3…, being the side length of the patch.

### 3.3. Dictionary Learning

To stabilize the sparse code, a *L*_2_-norm regularization term is added to form the elastic net [25, 26] for deriving the sparse code in the form of

(9) $$\begin{array}{c} \hat{\alpha }=\arg\underset{\alpha }{\min}\frac{1}{2}{\left\|\;s-D\alpha \;\right\|}_{2}^{2}+\lambda_{1}{\left\|\;\alpha \;\right\|}_{1}+\frac{\lambda_{2}}{2}{\left\|\;\alpha \;\right\|}_{2}^{2}, \end{array}$$

where *λ*_1_ and *λ*_2_ are, respectively, parameters to control sparsity and stability. Likewise, the dictionary learning objective function will be in the form of elastic net to replace the *L*_0_-norm or *L*_1_-norm.

(10) $$\begin{array}{c} \underset{\alpha ,D}{\min}\;\frac{1}{n}\left(\;\frac{1}{2}\overset{n}{\underset{i=1}{\sum }}{\left\|\;s_{i}-D\alpha_{i}\;\right\|}_{2}^{2}+\lambda_{1}{\left\|\;\alpha_{i}\;\right\|}_{1}+\frac{\lambda_{2}}{2}{\left\|\;\alpha_{i}\;\right\|}_{2}^{2}\;\right). \end{array}$$

For classifying voxels into infarct and noninfarct (normal), two subdictionaries are needed, which are denoted, respectively, as *D*_infarct_ and *D*_normal_.

**Figure 4:**
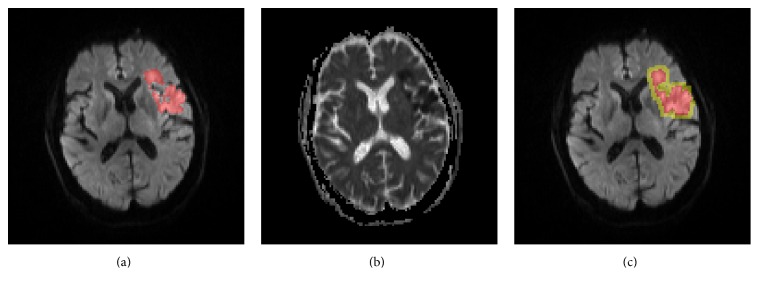
From (a) to (c), an axial slice of DWI with infarcts marked in red, the corresponding ADC, and the derived positive samples (in red) and negative samples (in yellow).

As the number of voxels of infarcts is far smaller than that of normal voxels, selection of training samples needs careful attention to achieve representative sampling while avoiding unbalanced samples. For training, the set of infarct voxels forms the positive samples and is denoted as GT(*x*, *y*, *z*). GT(*x*, *y*, *z*) is iteratively dilated with a structuring element of radius being 1 voxel until the dilated size is not smaller than the original size of GT(*x*, *y*, *z*). Those noninfarct voxels included in the dilated GT(*x*, *y*, *z*) are then acting as the negative samples. The rationale behind this procedure will be explained in Discussion.  Figure 4 shows the positive samples (in red) and negative samples (in yellow).

The objective function (formula (10)) could be optimized by an online algorithm based on stochastic approximations [27].

### 3.4. Derivation of Regions-of-Interest [23]

**Figure 5:**
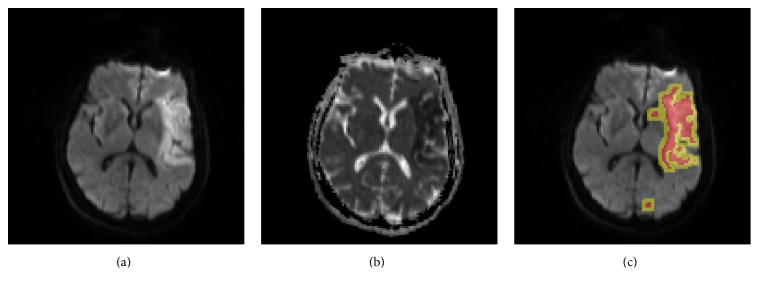
From (a) to (c), an axial slice of DWI and ADC and the corresponding ROIs for detecting infarction overlaid in color on the DWI, where regions in red are initial ROIs modified from [22] while those in yellow are from morphological dilation.

Due to the complexity and inhomogeneity of ischemic infarcts, there may be infarct-mimics in DWI volumes, which could be recognized as false positives. The ROIs are to include as many as possible infarct voxels and to exclude most infarct-mimics to reduce subsequent computational cost and enhance reliability. The ROIs are derived from dilation of the initial ischemic regions modified from [22] by thresholding ADC maps with constraints on DWIs. Specifically, denote the most frequent ADC value for all voxels within the brain mask as ADC_ref_, and any voxels with ADC(*x*, *y*, *z*) not greater than 0.75*∗*ADC_ref_ are checked to formulate connected components. Any connected components with average DWI values not smaller than the intensity average plus the intensity standard deviation of brain voxels on DWI at the corresponding axial slice are kept as part of region *R*_1_. Voxels with ADC value within (0.75*∗ADC*_ref_, 0.85*∗*ADC_ref_) are checked to formulate connected components and are added to *R*_1_ if the component has at least one neighboring voxel in *R*_1_. *R*_1_ is then increased by morphological dilation with a structuring element of radius *R*_*d*_. Suppose the *z* coordinates of *R*_1_ are in the range of *z*_0_ to *z*_*n*_ with *z*_*n*_ ≥ *z*_0_; then the *R*_1_ regions on the axial slice *z*_0_ and *z*_*n*_ are, respectively, pasted to axial slice *z*_0_ − 1 and *z*_*n*_ + 1 to attain the eventual ROIs.  Figure 5 shows an axial slice of DWI, ADC, and the corresponding ROIs (in color), where regions in red are initial ischemic infarcts and those in yellow are added voxels through the described morphological dilation.

### 3.5. Classification

Once ROIs are determined, voxels within the ROIs are classified as infarct or normal based on SRC according to patch features of voxels. The classification procedure is as follows:

 (1) A global dictionary *D* is formed by concatenating the two subdictionaries *D* = {*D*_infarct_, *D*_normal_}.
 (2) The sparse code $\hat{\alpha }$ of a new sample *s* is computed by optimizing the elastic net function in formula (9) with respect to the global dictionary *D*.
 (3) Compute the residue for each class
(11) $$\begin{array}{c} r_{i}\left(\;s\;\right)={\left\|\;s-D_{i}\delta_{i}\left(\;\hat{\alpha }\;\right)\;\right\|}_{2}^{2}, \end{array}$$
 where *i* = {infarct, normal} and *δ*_*i*_(·) is a characteristic function that would select the coefficients associated with class *i*.
 (4) Classification according to the residue of each class is as follows:
(12) $$\begin{array}{c} \text{Label}\left(\;s\;\right)=\arg\underset{i}{\min\;}r_{i}\left(\;s\;\right). \end{array}$$

## 4. Experiments

Tiantan Hospital and Tianjin Huanhu Hospital have been involved in a National Stroke Registry since 2005, which registered patients prospectively with stroke ictus within 6 hours according to a preestablished system [22]. The study included 98 consecutive patients (31 women and 66 men, age range 24–78 years) with confirmed ischemia. The protocol of the research has been approved by the Institutional Review Board of both hospitals. All patients gave written consent and provided permission for scientific and educational purpose.

The baseline DW imaging was carried out with two 3.0 Tesla scanners (Trio-Tim, Siemens, Erlangen, Germany) with a spin-echo, multislice, and single shot echo-planar imaging of *b* = 0 and 1000 or 1500 s/mm^2^ and the corresponding ADC map. The DWIs in this study are isotropic whose intensities are the cubic root of the multiplied signal intensities of the three individual images acquired with a diffusion gradient in each of the three orthogonal directions (*x*, *y*, and *z* axes). The imaging covered the whole brain, with 19–24 axial slices of a 5 mm slice thickness and 1–1.5 mm gap, most matrixes being 128 × 128 with few being 156 × 156 or 384 × 384 to have an in-plane resolution of 0.60 mm to 1.80 mm.

A neuroanatomy expert (QH) manually drew the infarction regions using in-house software (that could adjust contrast, enlarge, pan, and undo) as the reference for measuring the accuracy of automatic algorithms. When ischemia boundaries were not clear, both DWI and ADC were checked, and a neuroradiologist was invited for discussion (YZ) to make the drawing as accurate as possible.

The algorithm was implemented with C++ based on SPAMS [27]. All experiments were carried out on a Pentium 4 PC with 2.4 GHz CPU (4 cores) and 4G RAM. The segmentation performance was tested by 2-fold cross validation. All training datasets were randomly divided into 2 groups with equal number of datasets. One group was used as training set while the other was used as testing set and vice versa. To quantify the infarct segmentation, the following measures are adopted as used in other investigations [7, 8]: DC, sensitivity, specificity, positive prediction value (PPV), and negative prediction value (NPV) given below:

(13) DC=2×TPFP+2×TP+FN,sensitivity=TPTP+FN,specificity=TNTN+FP,PPV=TPTP+FP,NPV=TNTN+FN.

Here TP, TN, FP, and FN are, respectively, for true positive, true negative, false positive, and false negative.

The execution time for segmenting a dataset includes 5 seconds for determining ROIs and 1.9 seconds for classifying infarcts from ROIs on a Pentium 4 PC with 2.4 GHz CPU (4 cores) and 4 G RAM).

### 4.1. Parameters

Experiments are carried out to choose the appropriate parameters in dictionary learning and sparse representation in terms of DC. Best performance is achieved with *λ*_1_ = 0.3, *λ*_2_ = 0.1, *R*_*p*_ = 1, *R*_*d*_ = 2, and *K* = 200. Relevant experiments are conducted to see the dependency of accuracy on these parameters. When changing one or two parameters, the other parameters are fixed as those that attain the best performance.

**Figure 6:**
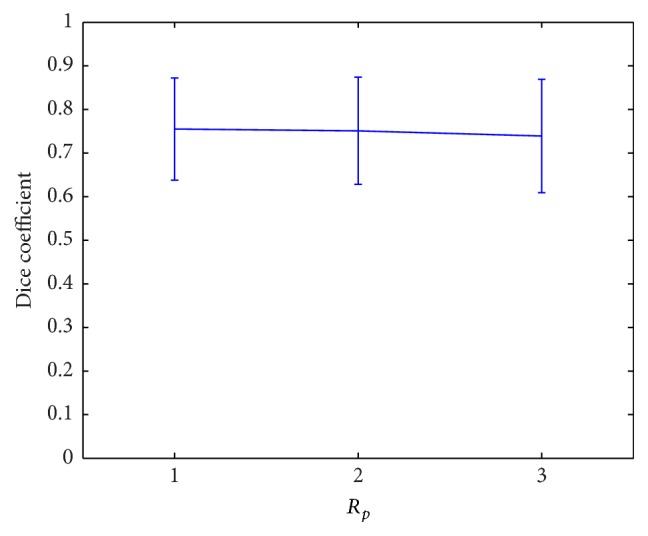
Classification with different patch sizes.

**Figure 7 fig7:**
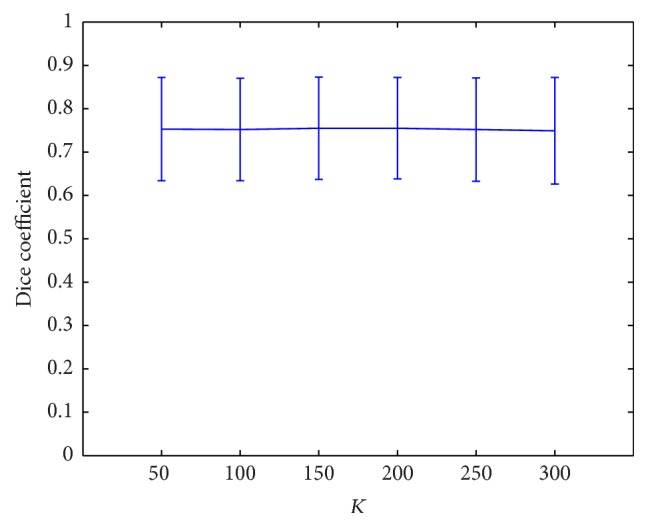
Classification with different dictionary sizes.

**Figure 8:**
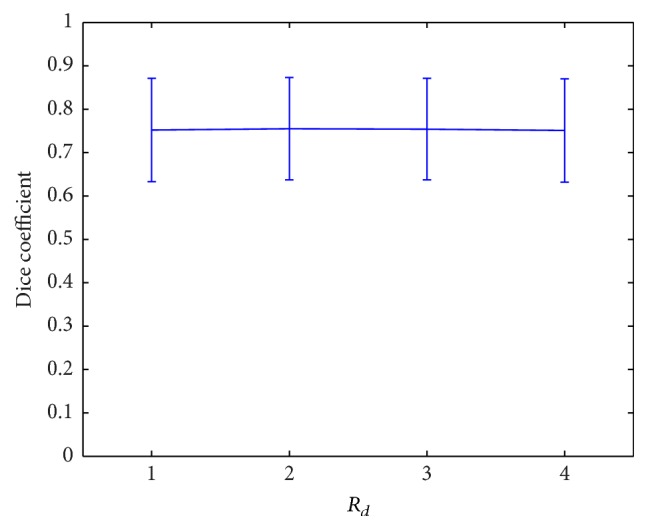
Classification with different *R*_*d*_.

The two parameters *λ*_1_ and *λ*_2_ are changed simultaneously, both taking one of the values of {0.1, 0.3, 0.5, 0.7, 0.9} (Table 1). Experiments of accuracy dependency on other parameters are carried out separately, with *R*_*p*_ to be one of the values of {1, 2, 3} (Figure 6), *K* taking a value of {50, 100, 150, 200, 250, 300} (Figure 7), and *R*_*d*_ being one of the values of {1, 2, 3, 4} (Figure 8).

### 4.2. Role of the Regions-of-Interest

We have conducted experiments to validate the effect of ROIs on ischemic lesion segmentation. To implement the proposed method without ROIs, two minor modifications have been made to the proposed method with ROIs. First, in addition to the negative samples of the proposed method, another equal portion of negative samples from the rest of the brain is included to represent the normal brain structure for learning the dictionary *D*_normal_. Second, after every voxel is classified by sparse representation classification as a candidate lesion voxel, a postprocessing step is applied. The postprocessing is to form candidate lesion region through connected component analysis and eliminate candidate lesion regions with less than 3 voxels, as well as eliminate candidate lesion regions with low average DWI (less than the most frequent value of DWI) or high average ADC (greater than the most frequent value of ADC). It was found that the proposed method without ROIs could yield a Dice coefficient of 0.673 ± 0.179, a sensitivity of 0.797 ± 0.168, and a specificity of 0.999 ± 0.001.

In case of taking the whole brain as the ROIs, the average execution time for segmenting a dataset is substantially increased from 7 seconds to 39 seconds.

### 4.3. Comparison with Existing Methods

The proposed method is to be compared with divergence measure based method (DM method) [7] and FCM method [8]. It is worth noting that the DC values reported in [7] (0.67) and [8] (0.90) are based on their data, and none of them used the data within 6 hours from ictus. As demonstrated in [7], data at admission are more difficult to be segmented due to substantial variability in appearance [6]. For a fair and relevant comparison, we have tried our best to implement the other two methods with minor enhancement to attain the best performance on the 98 datasets within 6 hours from symptom onset.

We are unable to use ROIs derived from Section 3.4 for FCM/DM because the two algorithms depend on the whole volume or slice for its calculation (either for histogram calculation (DM) of the two hemispheres and clustering into 50 clusters (FCM) (ROI may contain too few voxels to be categorized into 50 clusters)). In other words, it is very hard, if not impossible, to implement FCM/DM methods just for data within the ROIs. We have thought out and implemented one way to incorporate the ROIs for comparison: to segment the lesion using the original FCM/DM and remove those lesions outside the ROIs. To simplify denotations, these two implementations are, respectively, denoted as FCM_ROI and DM_ROI. Altogether there are 5 methods to be compared, namely, the proposed, DM, DM_ROI, FCM, and FCM_ROI methods.

**Figure 9:**
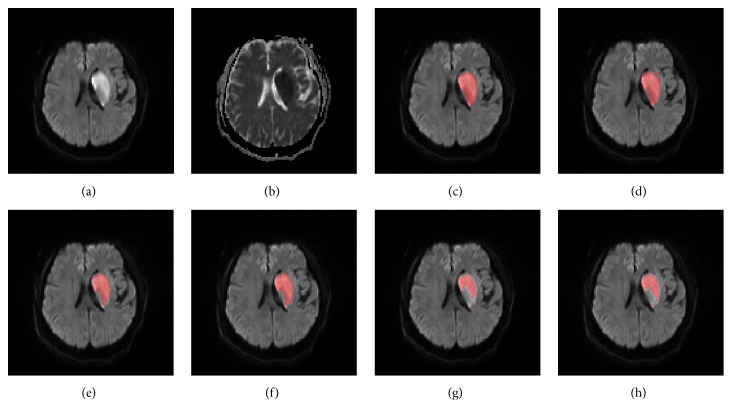
An axial slice of a patient with deep inhomogeneous infarcts, the DWI (a), ADC (b), ground truth infarcts (c), segmented infarcts by the proposed method (d), the FCM method (e), the FCM_ROI method (f), the DM method (g), and the DM_ROI method (h).

**Figure 10:**
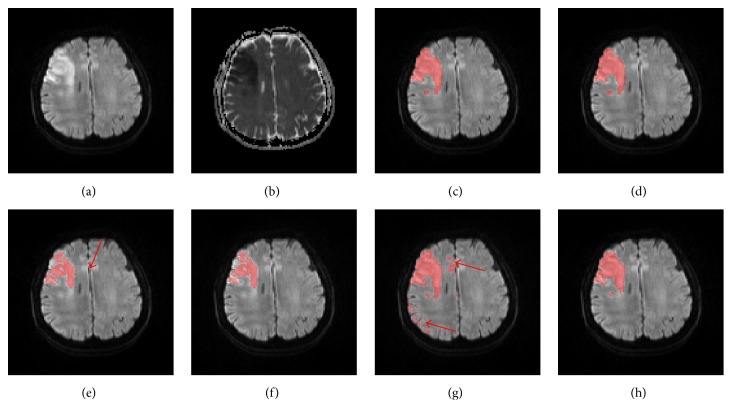
An axial slice of a patient with inhomogeneous infarcts involving the cortex, the DWI (a), ADC (b), ground truth infarcts (c), segmented infarcts by the proposed method (d), the FCM method (e), the FCM_ROI method (f), the DM method (g), and the DM_ROI method (h). Red arrows point to false positive regions, which could be eliminated by ROI confinement.

Figures 9 and 10 show, respectively, an axial slice with deep inhomogeneous infarcts and an axial slice with inhomogeneous infarcts involving the cortex, ground truth, and the segmented infarcts of the five methods.

**Figure 11:**
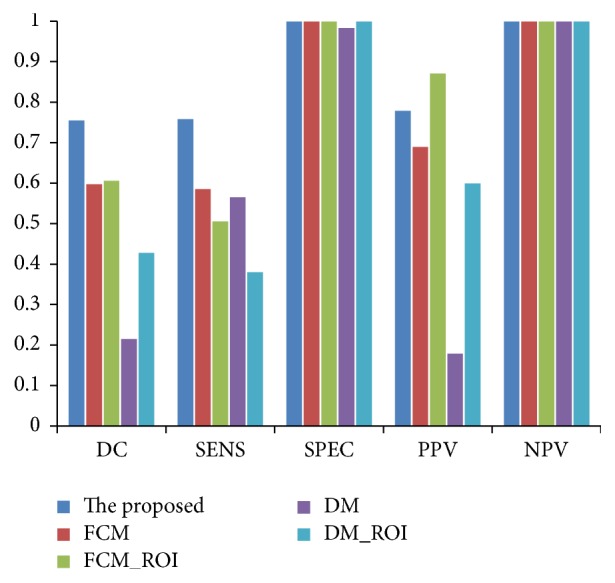
Performance comparison with existing methods. SEN for sensitivity, SPE for specificity, PPV for positive prediction value, and NPV for negative prediction value.

The segmentation performance of the 98 datasets by the proposed, FCM, FCM_ROI, DM, and DM_ROI methods in terms of DC, sensitivity, specificity, PPV, and NPV is summarized in Table 2 and shown in Figure 11.

## 5. Discussion

An SRC based method has been proposed and validated to segment infarcts from hyperacute ischemic stroke patient data. It consists of dictionary learning and classification. The first stage is carried out offline with elastic net. Then reconstruction residue is used for voxel-wise classification. Experiments are conducted to determine the appropriate parameters, including dictionary size *K*, *λ*_1_ to control the sparsity of the sparse code, *λ*_2_ to control the stability of the sparse code, radius of patches *R*_*p*_, and size of structuring element *R*_*d*_ to dilate the initial ischemic region for deriving the ROIs. The proposed method achieves best accuracy when these parameters are, respectively, 200, 0.3, 0.1, 1, and 2 with features being extracted from 2D patches. With different combinations, the accuracy DC could vary from 0.660 ± 0.163 to 0.755 ± 0.117 (Table 1). As the segmentation accuracy is sensitive to *λ*_1_ and *λ*_2_ (Table 1), they need to be determined with care. Once they are fixed, the segmentation is not sensitive to the variation of other parameters (Figures 6 [Fig fig7]–8). In other words, the algorithm is robust to parameters *K*, *R*_*p*_, and *R*_*d*_.

Experiments have been conducted to compare the performance of 2D patches and 3D patches which are used to train dictionary and sparse coding. Results show that the segmentation accuracy with 2D patches (DC = 0.755 ± 0.117) is slightly better than that with 3D patches (DC = 0.749 ± 0.123), which may imply that neighboring axial slices will add confusing information due to the large slice spacing.

In addition, we have carried out extra experiments to compare classification performance between elastic net and *L*_1_-norm constraints. For *L*_1_-norm constraints based on formulas (3) and (4), optimum *λ* and *K* are found through experiments to be, respectively, 0.9 and 150. For a fair comparison, both elastic net and *L*_1_-norm constraints are based on 2D patches of *R*_*p*_ being 1 and *R*_*d*_ being 2, with other parameters being optimum. As expected, elastic net yields higher DC than the *L*_1_-norm constraint (0.755 ± 0.117 versus 0.749 ± 0.119), which may imply that elastic net could better balance sparsity and stability of sparse codes than *L*_0_-/*L*_1_-norm at least for classification of ischemic infarcts. Gao and his colleague [14] were the first to advocate elastic net and showed similar difference to ours (difference between DC of elastic net and that of *L*_0_/*L*_1_-norm around 0.007).

Because ischemic infarcts are inhomogeneous and are undergoing variation at hyperacute stage, the boundaries between the infarcts and noninfarcts are usually blurred. We thus hypothesize that samples near the infarct boundaries are more difficult to be differentiated. To validate this assumption, another classification model is derived in a similar way to the proposed one with the only difference being that the negative samples are randomly picked from noninfarct voxels. As expected, the classification model from randomly picked negative sample yields significantly lower accuracy than the proposed one (DC 0.706 ± 0.121 versus 0.755 ± 0.118, *p* < 0.001 according to the paired *t*-test). This additional experiment justifies the way to pick up negative samples near the boundaries during training. As processing time is critical for hyperacute ischemic stroke data, ROIs are introduced to confine the classification space. Due to the substantial variability of DWI and ADC intensities of infarcts and artifacts with similar DWI and/or ADC intensities to infarcts, it is hard to determine appropriate candidate regions of infarcts or ROIs. For this purpose, we extend the ischemic regions calculated from our previous work that are based on decreased ADC with DWI being not low [22] that can include regions with unclear boundaries on DWI. The initial ROIs modified from [22] could yield an average DC of 0.601 ± 0.177, sensitivity of 0.689 ± 0.164, and specificity of 0.999 ± 0.001, being better than FCM method [8] and DM method [7]. After dilation and pasting the two extreme axial slices, the eventual ROIs contain most infarct voxels to have a sensitivity of 0.919 ± 0.105, which means that most infarct voxels not included in the initial ROIs are within the neighborhood. From the experiments on changing the neighborhood size *R*_*d*_ (Figure 8), an *R*_*d*_ of 2 attains best balance between inclusion of infarct voxels and exclusion of infarct-mimic voxels. The procedure to derive ROIs is reflected in the derivation of negative samples during training, that is, in the vicinity of positive samples through dilation and pasting. In the future, we will be working on derivation of ROIs with higher sensitivity and higher DC.

The experiments on taking the whole brain as the ROIs (Section 4.2) will yield lower Dice (0.673 versus 0.755), higher sensitivity (0.797 versus 0.758), and equal specificity (0.999) as compared with the proposed algorithm with ROIs. For the proposed method with ROIs, the lower sensitivity reflects the fact that the ROIs do not include all the ischemic lesions, while the higher Dice implies a net gain in accuracy to balance between excluding lesion mimics and missing some real lesions, as compared with the proposed method without the ROIs. We may thus argue that the introduction of ROIs could remove ischemic lesion mimics at the cost of excluding few ischemic lesions to have a net gain in accuracy, as well as speed up the segmentation substantially (from 39 seconds to 7 seconds, Section 4.2).

Quantification of infarcts from DW imaging at baseline within 6 hours from onset is crucial to guide treatment planning such as thrombolysis. As pointed out in [6], infarcts on DWI and ADC imaged within 6 hours are most difficult to be delineated by experts due to substantial variability in intensities and ill-defined edges as compared with those imaged after 6 hours. As the DM method [7] is basically a global thresholding method to determine the DWI threshold based on divergence measure, it is not appropriate for processing data imaged within 6 hours due to the substantial variability in intensities. For the FCM method [8], it is dependent on the prominent edge on DWI for confirmation of infarcts, which may be the case for data within 10 days. As the data in this study are all within 6 hours from ictus, the ischemic lesion is still evolving and may have ill-defined edges on DWI; it is understandable that FCM will have a bad performance for these ischemic data. Derivation of ROIs from Section 3.4 is a way to incorporate prior knowledge to differentiate between ischemic lesions and ischemic lesion mimics based on our previous work [22]. The ROIs are part of the proposed method to enhance the segmentation accuracy from an average DC of 0.673 (Section 4.2, the proposed method without ROIs) to an average DC of 0.755 (Table 2). When the DM and FCM methods are confined by the ROIs as illustrated in Section 4.3, the DM_ROI and FCM_ROI could yield a higher DC (being, resp., 0.417 and 0.606, Table 2), which are still significantly lower than the proposed method (*p* < 0.001). The proposed method could cope with the variability in intensities and ill-defined edges of DWI and ADC data imaged within 6 hours through learning the pattern from training samples. As such, the proposed method attain significantly higher accuracy in terms of DC, sensitivity, and PPV than [7, 8] all with *p* < 0.001, while there exists no significant difference for the specificity and FPV among the three methods (Table 2). The superior performance of the proposed method may be ascribed to the following characteristics of the method.

**Figure 12:**
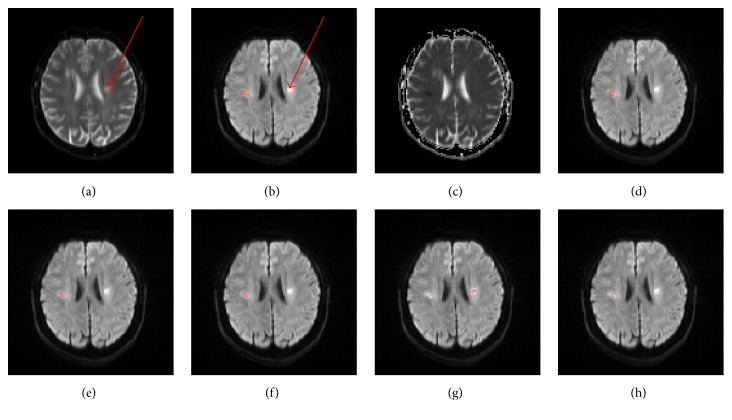
Illustration of the shine-through artifact on DWI (arrow) of an axial slice, B0 (a), DWI (b), ADC (c), segmented infarcts by the proposed method (d), FCM method (e), FCM_ROI method (f), DM method (g), and DM_ROI method.

First, it takes into account B0, DWI, ADC, and asymmetry with respect to the MSP, so the artifacts on DWI due to shine-through effect could be eliminated (Figure 12(d)). For the FCM method [8], it imposes ADC constraint to confine infarcts so the shine-through artifact could be removed (Figure 12(e)). As for the DM method [7], it could not get rid of shine-through artifact because it is purely based on DWI (Figure 12(g)). Both DM_ROI and FCM_ROI could handle the shine-through artifact due to the introduction of ROI constraints.

**Figure 13:**
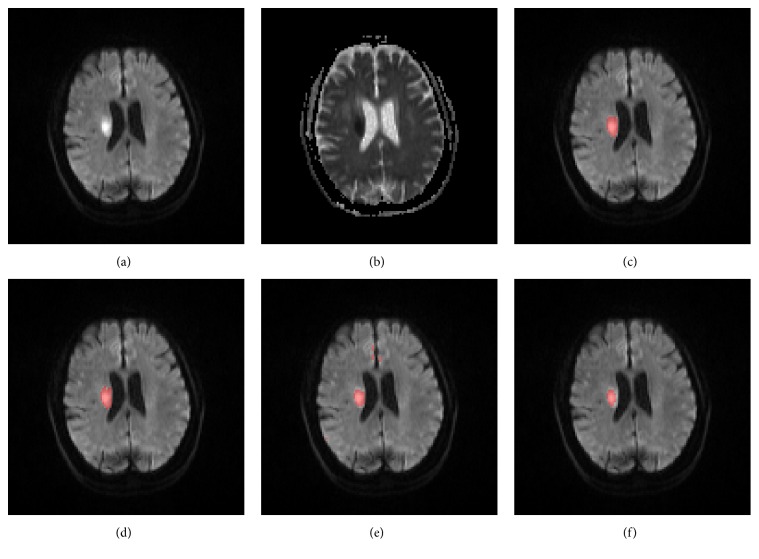
Illustration of the intensity variation of infarcts of an axial slice, DWI (a), ADC (b), ground truth infarcts (c), segmented infarcts by the proposed method (d), FCM method (e), and FCM_ROI method (f).

Second, the proposed method could handle better than [7, 8] for infarcts with intensity variability on DWI, as it is based on learning samples with intensity variation.  Figure 13 shows an axial slice with lower intensities around the infarct border on DWI; as such, the FCM [8] and FCM_ROI methods could not include the border (Figures 13(e) and 13(f)) while the proposed method could (Figure 13(d)). The DM method [7] fails to segment infarcts at this axial slice as it has a lower DWI intensity than infarcts at other axial slices.

**Figure 14:**
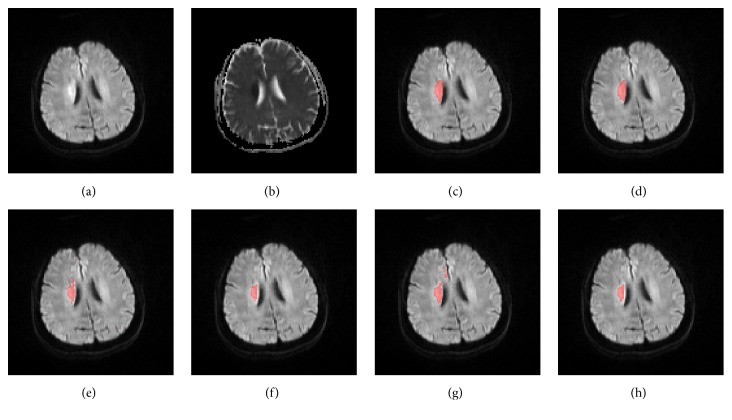
Illustration of ill-defined edges on DWI of an axial slice. DWI (a), ADC (b), ground truth (c), segmented infarcts by the proposed method (d), FCM method (e), FCM_ROI method (f), DM method (g), and DM_ROI method (h).

Third, the proposed method could handle better than [7, 8] infarcts with ill-defined edges on DWI, once again due to the fact that the delineation is based on learning samples with similar edges (Figure 14).

Fourth, introduction of the asymmetry map has significantly enhanced the segmentation accuracy. When only B0, DWI, and ADC are employed for SRC based training and classification, the best performance is DC = 0.681 ± 0.160 and sensitivity = 0.709 ± 0.188. When the asymmetry map is added, the DC and sensitivity have been, respectively, increased to 0.755 ± 0.118 and 0.758 ± 0.149. We also carried out experiments to segment based on thresholding the asymmetry map to find that the highest Dice achieved is 0.482 ± 0.233 with a sensitivity of 0.528 ± 0.220 and specificity of 0.999 ± 0.001 when the asymmetry threshold is around 40. As the performance based on thresholding the asymmetry map is substantially inferior to the proposed method, we may argue the following: (1) both infarcts and noninfarcts could cause asymmetry (sensitivity greater than 0 and specificity smaller than 1); (2) not all infarcts could be detected by asymmetry map (sensitivity is always smaller than 1); and (3) the proposed sparse learning framework is better than simple thresholding, and there is much complementary information from B0, DWI, and ADC for segmenting the infarcts (recall that the training of dictionaries is from the asymmetry map, DWI, ADC, and B0, Section 3.2).

The proposed method combines the advantages of SRC and our previous work to delineate infarcts based on DWI and ADC [22]. In particular, SRC could help to find sophisticated object patterns (such as infarcts with intensity variation and ill-defined edges), while the ROIs derived from [22] will confine the infarcts within candidate regions to decrease computational cost and exclude infarct-mimics. According to [28], a DC of 0.7 and above indicates a good agreement. As the proposed method could achieve good agreement (DC > 0.70) with speed (within 7 seconds), it could be a potential tool to be used clinically for guiding thrombolytic therapy.

The proposed method has only been validated on Siemens 3T scanners of two hospitals. It is our intension to design different dictionaries for different scanners to account for variability of imaging hardware. We are in the process of designing classifiers for GE scanners.

**Figure 15:**
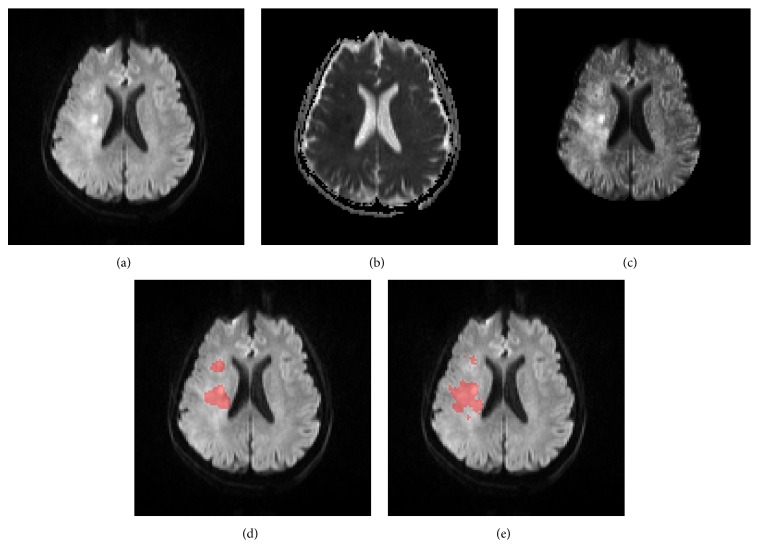
One axial slice with complex image properties. DWI (a), ADC (b), DWI + (DWI-ADC) (c), ground truth (d), and segmented infarcts by the proposed method (e). FCM, FCM_ROI, DM, and DM_ROI methods fail to segment infarcts at this axial slice.

The hyperacute ischemia sometimes exhibits substantial variability that are hard to be modeled mathematically, which is the major cause of deviation from ground truth infarcts of the proposed method. For these cases, manual delineation of infarcts is difficult and is based on experience, anatomical knowledge, and comprehension of the DWI and ADC. To aid manual delineation, a new volume, that is, DWI(*x*, *y*, *z*) + (DWI(*x*, *y*, *z*) − ADC(*x*, *y*, *z*)), is created, which is similar to DWI but emphasizes infarcts.  Figure 15 shows an axial slice with complex image properties and the segmentation of the proposed method (the FCM, FCM_ROI, DM, and DM_ROI methods fail to segment). New tools and methods are yet to be developed for better segmentation of infarcts with complicated imaging features that are even hard to be manually delineated by human experts.

## 6. Conclusion

In this paper, an SRC based cerebral infarct segmentation method is explored and validated against 98 ischemic datasets scanned within 6 hours from ictus. The proposed method could handle well infarcts with intensity variability and ill-defined edges to yield significantly higher DC (0.755 ± 0.118) than the FCM method [8] (0.597 ± 0.204, *p* < 0.001) and DM method [7] (0.215 ± 0.213, *p* < 0.001) and their enhanced versions by confining their segmentations within the ROIs (0.606 ± 0.201, *p* < 0.001; 0.428 ± 0.342, *p* < 0.001). It could segment infarcts of a patient from baseline DW imaging within 7 seconds on a Pentium 4 PC with 2.4 GHz CPU (4 cores) and 4 G RAM. The superior performance is mainly ascribed to the comprehensive inclusion of the DW imaging and introduced asymmetry map, learning based nature that could learn complex infarct patterns, adoption of elastic net to stabilize sparse code, and introduction of ROIs to speed up the classification procedure as well as exclude lesion mimics. The proposed method could provide a potential tool to quantify infarcts from DW imaging at hyperacute stage with accuracy and speed to assist the decision making especially for thrombolytic therapy.

## Figures and Tables

**Table 1:** Dice coefficients with different combinations of *λ*_1_ and *λ*_2_.

*λ*_2_	*λ*_1_				
	0.1	0.3	0.5	0.7	0.9
0.1	0.737 ± 0.129	**0.755** ± **0.117**	0.754 ± 0.119	0.745 ± 0.125	0.716 ± 0.133
0.3	0.744 ± 0.126	0.735 ± 0.127	0.728 ± 0.135	0.733 ± 0.134	0.738 ± 0.125
0.5	0.724 ± 0.132	0.705 ± 0.144	0.711 ± 0.142	0.722 ± 0.137	0.738 ± 0.128
0.7	0.689 ± 0.148	0.684 ± 0.154	0.694 ± 0.147	0.713 ± 0.137	0.736 ± 0.128
0.9	0.660 ± 0.163	0.658 ± 0.163	0.673 ± 0.154	0.704 ± 0.140	0.731 ± 0.130

**Table 2:** Segmentation performance comparison between the proposed and four other methods.

Methods	DC	SEN	SPE	PPV	NPV
Proposed	0.755 ± 0.118	0.758 ± 0.149	0.999 ± 0.001	0.779 ± 0.141	0.999 ± 0.001
FCM	0.597 ± 0.204	0.585 ± 0.221	0.999 ± 0.001	0.689 ± 0.230	0.999 ± 0.001
FCM_ROI	0.606 ± 0.201	0.505 ± 0.215	0.999 ± 0.001	0.871 ± 0.156	0.999 ± 0.001
DM	0.215 ± 0.213	0.565 ± 0.346	0.983 ± 0.024	0.179 ± 0.200	0.999 ± 0.001
DM_ROI	0.428 ± 0.342	0.380 ± 0.321	0.999 ± 0.001	0.599 ± 0.404	0.999 ± 0.001

SEN for sensitivity, SPE for specificity, PPV for positive prediction value, and NPV for negative prediction value.
