# Corrigendum to “Segmentation of Hyperacute Cerebral Infarcts Based on Sparse Representation of Diffusion Weighted Imaging”

**DOI:** 10.1155/2018/8092523

**Published:** 2018-11-05

**Authors:** Xiaodong Zhang, Shasha Jing, Peiyi Gao, Jing Xue, Lu Su, Weiping Li, Lijie Ren, Qingmao Hu

**Affiliations:** ^1^Shenzhen Institutes of Advanced Technology, Chinese Academy of Sciences, 1068 Xueyuan Boulevard, Shenzhen 518055, China; ^2^University of Chinese Academy of Sciences, Beijing, China; ^3^Beijing Tiantan Hospital, Capital Medical University, 6 Tiantan Xili, Beijing 100050, China; ^4^Shenzhen Second People's Hospital, 3002 West Sungang Road, Shenzhen 518035, China

In the article titled “Segmentation of Hyperacute Cerebral Infarcts Based on Sparse Representation of Diffusion Weighted Imaging” [1], there was a missing affiliation for the first and last authors. The corrected authors' list and affiliations are shown above.
